# Eligibility criteria for pediatric patients who may benefit from anti SARS-CoV-2 monoclonal antibody therapy administration: an Italian inter-society consensus statement

**DOI:** 10.1186/s13052-021-01187-1

**Published:** 2022-01-12

**Authors:** Marcello Lanari, Elisabetta Venturini, Luca Pierantoni, Giacomo Stera, Guido Castelli Gattinara, Susanna Maria Roberta Esposito, Silvia Favilli, Emilio Franzoni, Eleonora Fusco, Paolo Lionetti, Claudio Maffeis, Gianluigi Marseglia, Laura Massella, Fabio Midulla, Alberto Zanobini, Marco Zecca, Alberto Villani, Annamaria Staiano, Luisa Galli, Other Collaborators, Francesco Blasi, Angelo Di Giorgio, Daniele Donà, Amelia Licari, Massimo Martinelli, Antonio Mastrangelo, Michele Miraglia del Giudice, Giangiacomo Nicolini, Fabrizio Pugliese, Pasquale Striano, Giuliana Valerio

**Affiliations:** 1 Pediatric Emergency Unit, Scientific Institute for Research and Healthcare (IRCCS), Azienda Ospedaliero-Universitaria di Bologna, Bologna, Italy; 2 Italian Association of Children’s Hospital (AOPI), Rome, Italy; 3 Infectious Diseases Unit, Meyer Children’s University Hospital, Florence, Italy; 4 Postgraduate School of Pediatrics, University of Bologna, Bologna, Italy; 5 Institute of Child Health, Ospedale Bambino Gesù – IRCCS, Rome, Italy; 6 Pediatric Clinic, Pietro Barilla Children’s Hospital, Department of Medicine and Surgery, University of Parma, Parma, Italy; 7 Cardiology Unit, Meyer Children’s University Hospital, Florence, Italy; 8 Department of Medical and Surgical Sciences, University of Bologna, Bologna, Italy; 9 Postgraduate School of Pediatrics, University of Florence, Meyer Children’s Hospital, Florence, Italy; 10 Gastroenterology Unit, NEUROFARBA Department, University of Florence, Meyer Children’s Hospital, Florence, Italy; 11 Pediatric Clinic B, Mother and Child Hospital, Department of Surgery, Dentistry, Paediatrics, and Gynaecology, University of Verona, Verona, Italy; 12 Department of Pediatrics, University of Pavia, San Matteo Foundation IRCCS Policlinico, Pavia, Italy; 13 Division of Nephrology, Department of Pediatric Subspecialties, Bambino Gesù Children’s Hospital, IRCCS, Rome, Italy; 14 Department of Maternal Infantile and Urological Sciences, Sapienza University of Rome, Rome, Italy; 15 Pediatric Hematology/Oncology, Fondazione IRCCS Policlinico San Matteo, Pavia, Italy; 16 General Pediatrics Unit, Pediatric Emergency and General Pediatrics Department, Bambino Gesù Children’s Hospital, IRCCS, Rome, Italy; 17 Department of Translational Medical Science, Section of Pediatrics, University of Naples “Federico II”, Naples, Italy; 18 Department of Health Sciences, University of Florence, Florence, Italy

**Keywords:** Monoclonal antibody, COVID19, Adolescents, Risk factors

## Abstract

The fast diffusion of the SARS-CoV-2 pandemic have called for an equally rapid evolution of the therapeutic options.

The Human recombinant monoclonal antibodies (mAbs) have recently been approved by the Food and Drug Administration (FDA) and by the Italian Medicines Agency (AIFA) in subjects aged ≥12 with SARS-CoV-2 infection and specific risk factors.

Currently the indications are specific for the use of two different mAbs combination: Bamlanivimab+Etesevimab (produced by Eli Lilly) and Casirivimab+Imdevimab (produced by Regeneron).

These drugs have shown favorable effects in adult patients in the initial phase of infection, whereas to date few data are available on their use in children.

AIFA criteria derived from the existing literature which reports an increased risk of severe COVID-19 in children with comorbidities. However, the studies analyzing the determinants for progression to severe disease are mainly monocentric, with limited numbers and reporting mostly generic risk categories.

Thus, the Italian Society of Pediatrics invited its affiliated Scientific Societies to produce a Consensus document based on the revision of the criteria proposed by AIFA in light of the most recent literature and experts’ agreement.

This Consensus tries to detail which patients actually have the risk to develop severe disease, analyzing the most common comorbidities in children, in order to detail the indications for mAbs administration and to guide the clinicians in identifying eligible patients.

## Introduction

The fast diffusion of the SARS-CoV-2 pandemic together with the increasing knowledge of the virus have called for an equally rapid evolution of the therapeutic options evaluated to deal with this new pathogen. During the last two years, the scientific community has made an unprecedented effort investing enormous resources in the development of new therapies and vaccines against SARS-CoV-2. Human recombinant monoclonal antibodies (mAbs) have recently been approved by the Food and Drug Administration (FDA) for the emergency use in subjects infected with SARS-CoV-2 and with specific risk factors [1–3].

The Italian Medicines Agency (*Agenzia Italiana del Farmaco -* AIFA), in compliance with the decree of Health Ministry of 02-06-2021 (published on *Gazzetta Ufficiale* – GU – n. 32 of 02/08/2021), established the indications for use of the following mAbs: bamlanivimab (published on the GU n.58 of the 03/09/2021, revoked on the 05/06/2021 with notification on the GU n.108 of the 7/5/2021), bamlanivimab and etesevimab (published on GU n.66 of the 03/17/2021, last updated on GU n. 142 of the 06/16/2021) and casirivimab and imdevimab (published on GU n.71 of the 03/23/2021, last updated on GU n.187 of the 08/06/2021).

Currently the indications are specific for the use of two different mAbs combinations:

- Bamlanivimab+Etesevimab (produced by Eli Lilly)
- Casirivimab+Imdevimab (produced by Regeneron).

Both those drugs’ combinations act in the same way targeting epitopes of the Receptor Binding Domain (RBD) of the SARS-CoV-2’s spike protein, which represents the main antigen of the virus*.* This prevents the connection between the pathogen and the Angiotensin Converting Enzyme type 2 (ACE-2) receptors, blocking the spread into the host’s cells and causing the opsonization of the virus [4, 5].

The rapid development of these new molecules has been accompanied by poor data on their utilization in adults and by a lack of studies regarding efficacy and safety in pediatric patients. Notably, some studies in vitro have showed a limitation in control of replication of the viral variants and a growing risk of developing new variants [6, 7].

However, despite the limited data regarding these drugs, those seem to guarantee a favorable security and efficacy profile in adult patients [8] in the initial phase of infection (with mild or moderate symptoms and high viral loads) for which we still need a reliable therapeutic option. To date no studies about the efficacy and the safety of anti SARS-CoV-2 monoclonal antibodies in pediatric patients have been published. In addition, infected pediatric patients have often showed a favorable outcome regardless of the presence of risk factors or comorbidities [8]. For these reasons, their utilization is still under debate [9] calling for a judicious and detailed use.

Regarding these considerations and the risk of an increased diffusion of COVID-19, as Italian Society of Pediatrics (*Società Italiana di Pediatria -* SIP) and other pediatric subspecialist scientific society, we try to detail the indications for the use of mAbs in the pediatric population, upon the available evidence from the scientific literature and experts’ recommendation.

Therefore, FDA and AIFA have allowed those drugs to be used also in the pediatric patients limited to subjects aged at least 12 years, with recently diagnosed SARS-CoV-2 infection and mild or moderate symptoms, in presence of specific risk factors for an higher risk of progression to severe disease [10].

**AIFA’s indications** for the use of anti-SARS-CoV-2 mAbs (Bamlanivimab-Etevesimab o Imdevimab-Casarivimab) are reported in Table 1 [11].

**Table 1:** AIFA’s indications for the use of anti-SARS-CoV-2 mAbs in subjects between 12 and 17 years old

Patients aged ≥12 years old, who tested positive for SARS-CoV-2, with mild-moderate disease, a recent onset (less than 10 days, except for patients with immunodeficiency and persistently positive molecular test but negative serologic test)* and the presence of risk factors for severe disease: - Body Mass Index (BMI) ≥95°percentile for age and gender - Chronic renal failure, including hemodialysis or peritoneal dialysis - Uncontrolled Diabetes mellitus (HbA1c > 9% or 75 mmol/mol) or with chronic complications - Primary and secondary Immunodeficiencies - Haemoglobinopathies - Cerebral vascular diseases (including high blood pressure with organ damage) - Neurodevelopment and degenerative diseases - Chronic obstructive pulmonary disease e/o other respiratory chronic conditions (for examples asthma, pulmonary fibrosis or condition requiring oxygen therapy not for SARS-CoV-2) - Chronic hepatopathy (with following box warning: “mAbs have not been studied in patients with moderate or severe hepatic impairment”) *Bamlanivimab+Etesevimab is approved for outpatients (not-hospitalized patients) without supplemental oxygen therapy for COVID 19; Casirivimab+Imdevimab is approved also for hospitalized patients and for patients with conventional oxygen therapy (excluding high flow and mechanical ventilation)	

Those risk categories are derived from the existing literature which reports an increased risk for severe COVID-19 in children with comorbidities. However the studies analyzing the determinants for progression to severe disease are mainly monocentric, with limited numbers and reporting mostly generic risk categories (e.g. hearth diseases, chronic respiratory diseases, neurologic anomalies, immunodeficiencies) [12].

In particular, in a meta-analysis on 285,000 pediatric patients infected by SARS-CoV-2, children with comorbidities had a relative risk ratio of severe infection and mortality respectively of 1.79 and 2.81 more than those without comorbidities. Nevertheless, the relative low numbers of the children with the different comorbidities precluded an analysis of the relative risk (RR) for single underlying condition, except for obesity [13].

## Methods

This paper has been drawn up by the SIP on initiative of the *Pediatric Pharmacology Study Group* and the Italian Society of Pediatric Infectious Diseases (SITIP), in collaboration with the scientific committees and the experts of the main scientific societies affiliated to SIP (Italian Society of Pediatric Endocrinology and Diabetology – SIEDP; Italian Association of Pediatric Haematology and Onchology – AIEOP; Italian Society of Pediatric Cardiology – SICP; Italian Society of Infantile Respiratory Diseases – SIMRI; Italian Society of Pediatric Gastroenterolgy, Hepatology and Nutrition – SIGENP; Italian Society of Pediatric Allergology and Immunology – SIAIP; Italian Society for Pediatric Nephrology – SINePe; Italian Society of Pediatric Neurology – SINP; Technical table for Infectious Diseases of the Italian Society of Pediatrics; Italian Society of Cystic Fibrosis – SIFC).

PubMed database was searched from inception to the 31st July 2021 for papers on risk factors for progression to severe disease following COVID-19 infection. Papers describing the risk of infection, the course of disease and the outcome in the patients with the underlying conditions below were included. Due to the lack of evidence regarding the pediatric patients no age restrictions were applied to the search.

### Obesity

Obesity **(**defined by **BMI** ≥ **95° percentile by WHO)**, as described for the adult and in the only meta-analysis about pediatric patients, could be regarded as an important risk factor for the progression to severe/critic disease (RR: 2.87; 95%CI: 1.16–7.07) [13]. This statement is further supported by a recent multicenter study where obesity was found to be strongly associated to the risk of hospitalization (RR: 3.07; 95%CI: 2.66–3-54) [14].

The relation between obesity and severe disease outcome may due to the pro-inflammatory action of the adipose tissue with a local and systemic increase in cytokines as Interleukin-6 (IL-6). Other determinants are the endothelial disfunction, insulin resistance, renal damage and hypertension which are more frequent in the obese patient, even if more typical of the adult instead of the pediatric population [13, 15].

### Hematological risk factors

Patients with **sickle cells anemia** should be regarded as “high risk population”, because of their immunity disfunction secondary to the functional asplenia, the systemic vasculopathy and the increased thrombotic risk. In contrast, the limited data available for this disease in pediatric age describe for those patients a risk of progression to severe SARS-CoV-2 similar to that of the general population [16, 17].

However, given the limited quality of the papers available, mainly observational studies or case series, the use of mAbs in this category should still be considered.

About **thalassemia,** currently there are no studies about the pediatric age. In the adult patient the risk of developing a severe/critical infection is considered dependent on the severity of the hemoglobinopathy as affirmed in the position statement of the Thalassemia International Federation. This consensus identifies as exposed to a greater risk patients with severely reduced levels of hemoglobin (< 7 g/dl), severe comorbidities or iron overload [18, 19].

However, thalassemia itself, independently from hemoglobin levels, iron overload or other comorbidities carries a risk for thrombosis and for pulmonary complications which is greater than that of the general population [20, 21]. So this Panel retain reasonable allowing the use of anti-SARS-CoV-2 mAbs in patients with *thalassemia major* (transfusion-dependent thalassemia) or *intermedia* (non transfusion-dependent thalassemia), regardless of the hemoglobin levels (which may be influenced by the transfusions), iron overload or comorbidities.

For the same reason, in analogy to what observed in the adult population, the mAbs should be used also in patients with **hereditary or acquired thrombophilia** which are likely to be at greater risk to develop severe disease when infected by SARS-CoV-2.

Regarding patients with **onco-hematological** conditions, an in-press paper identifies lymphopenia (< 300/mmc), neutropenia (< 500/mmc) and high intensity treatment (Acute Monoblastic Leukemia [AML], induction and reinduction phase for patients with Acute Lymphoblastic Leukemia [ALA] and Non-Hodgkin Lymphoma [NHL], patients who undergone autologous [< 30 days] or allogenic [< 100 days] hematopoietic cells transplantation) as factors potentially connected with severe COVID-19 disease. In these categories of patients the administration of mAbs should thus be considered.

About **immunosuppressant therapies** (e.g. rituximab, anti-thymocyte globulin), the Panel considers reasonable a case-by-case approach under the guidance of the Specialized Pediatrician in charge for the patient.

### Transplant recipients

In **solid organ transplant** recipients, evidence from the adult suggests an increased mortality among those infected by SARS-CoV-2, which seems to be mainly secondary to the comorbidities and to the old age of these patients than to the transplant-related immunosuppression [22].

On the contrary, data regarding pediatric transplant recipient describes a severity of SARS-CoV-2 disease as in general population [23–25]. However, waiting for new data, the Panel retains appropriate to consent the use of mAbs in solid organ transplant recipients.

**Hematopoietic cells transplantation** recipients (within 3 months if autologous, 6 months if allogenic, or with chronic active *graft-*versus*-host-disease*) should be considered at high risk for progression to severe/critical disease if infected by SARS-CoV-2 as demonstrated by an important USA-based study [26].

Those patients are thus to prioritize in mAbs administration.

### Hearth conditions

It should be appropriate to consider **congenital heart diseases** as potential predictors for a negative *outcome* in subjects infected by SARS-CoV-2. In fact, viral infections may cause a sudden worsening in patients suffering from congenital heart diseases. Moreover, those subjects are often affected by other comorbidities, mostly respiratory, which can increase the risk burden. These considerations are further reinforced by studies analyzing SARS-CoV-2 infection in patients with congenital heart disease, which confirmed an enhanced risk for progression to severe disease [14, 27].

However, Scientific Societies have proposed a different sub-classifications, in order to identify the congenital heart diseases at higher risk [28, 29]. In these classifications, patients with congenital heart disease are considered at high risk if belonging to one (or more) of the following categories:

- single ventricle patients or patients who have undergone Fontan intervention (total cavo-pulmonary connection);
- severe valvopathy;
- chronic cyanosis (SpO2 < 85%);
- therapy-dependent cardiomyopathies;
- pulmonary hypertension on treatment;
- heart-transplant recipients;
- congenital heart diseases with significative comorbidities (e.g. chronic kidney disease, chronic pulmonary disease) [30].

### Respiratory conditions

Patients with **cystic fibrosis** (CF) could be considered as high risk subjects in case of infection by SARS-CoV-2 because of the general respiratory involvement and the greater susceptibility to infections. However, literature data seems to indicate as key determinants for severe disease the respiratory functionality tests results during the last year, the nutritional status (in particular a reduced BMI) and the transplant if received, rather than CF itself [31–33].

The Italian Society of Infantile Respiratory Diseases *(Società Italiana Malattie Respiratorie Infantili* - SIMRI) underlines that this infection in CF patients is mostly mild, even if a close follow-up remains of pivotal importance in order to exclude long term complications [34].

The Panel retains appropriate to extend mAbs utilization to patients with CF and moderate-severe respiratory disfunction, reduced BMI, transplant-recipients or with important comorbidities, because of the potential risk of complications CF-related.

Concerning **asthma**, it should be reasonable, following the periodic updates of Global Initiative for Asthma (GINA), to make a distinction. Patients with mild-moderate (well controlled) asthma doesn’t seem to be at higher risk of unfavorable outcome in case of COVID-19, while severe asthma (on chronic drug therapy with high-dosage of inhaled steroids or on specific mAbs, in need of oral steroid therapy to control symptoms or hospitalized for severe asthma) carries an enhanced risk [35–38].

In a literature review made by the SIMRI, an increased risk is not reported for patients with asthma, but the evidence is limited and fragmented. Considering all these considerations, it should be useful to limit mAbs use to patients with uncontrolled or severe asthma [39].

For other diseases affecting the respiratory system such as bronchopulmonary dysplasia, bronchiolitis obliterans, pulmonary fibrosis, interstitial lung disease (ILD) and chronic pulmonary graft-versus-host-disease (GVHD), there are currently little data supporting an higher risk of severe disease upon infection from SARS-CoV-2. Still, considering the severity of these conditions the Panel retain appropriate to consent the use of mAbs in these patients. Likewise, the **carriers of tracheostomy** should be considered as high risk subjects and thus to prioritize in the mAbs administration, even if the augmented risk for progression to severe disease seems to be depend more from their comorbidities (especially neurologic) than by the presence of the device itself [40–42].

For **primary ciliary dyskinesia** and **bronchiectasis,** there are currently no studies describing any enhanced risk for severe COVID-19. Data from the adult seems to support only a mildly increased risk for disease progression in patients suffering from these conditions when compared to the general population [43]. In addition, a prospective study in adults show a less severity and less incidence of COVID-19 in this category of patients than general population [44]. Considering the fragmented and contrasting evidence available, it is reasonable to allow the use of mAbs in those patients, leaving a more precise and individualized decision to the Pediatrician in charge for the patient.

### Inflammatory bowel diseases (IBDs)

Patients with **IBDs** according to the data recently published by both the SECURE-IBD *Consortium* and the Pediatric IBD Porto Group (ESPGHAN) don’t seem to be at higher risk to develop severe SARS-CoV-2 infection. On the other hand, preliminary findings have shown how immunosuppressants as infliximab used for the treatment of IBDs, in particular when administered in combination with an immune-modulator, could reduce anti-SARS-CoV-2 vaccines immunogenicity [45, 46]. Therefore, on the basis of the overall greater vulnerability of this class of patients, driven by chronic inflammation, immunosuppressant therapies and reduced vaccines’ effectiveness, the Panel deems appropriated including IBDs patients in the categories with access to anti SARS-CoV-2 mAbs [45–47].

### Liver disease

Considering the pediatric population affected by **liver disease,** a recently published revision remarks how have still not been identified subcategories to be regarded as at higher risk for severe disease [48]. An Italian study has outlined as the overall health of the children with chronic liver disease has remained stable during the first COVID-19 outbreak in March 2020 [49]. These findings are further reinforced by other studies focused on that issue, describing how children with chronic liver conditions, even if on immunosuppressant drugs (notably those who undergone liver transplantation or affected by autoimmune liver disease) are not at high risk of severe disease [50–53]. Hence, upon the available evidence the Panel retain appropriate not to include children affected by chronic liver diseases among those eligible to mAbs treatment.

### Immunodeficiencies

According to the recent European Society for Immunodeficiencies (ESID) statement, there is still no evidence in support of a different risk for severe disease in immunocompromised or with primary immune deficit patients as compared to the general population [54–56].

In fact, being immunosuppressed seem to be less influential than other comorbidities in conditioning the outcome of SARS-CoV-2 infection in pediatric age. Among patients with primary immunodeficiencies (PID), older age and organ impairment (chronic pulmonary disease, heart disease, renal or liver failure) in analogy to what observed in the general population, appear to play a more relevant role in conditioning the prognosis as compared to the immunosuppression itself [54, 55, 57–63]. In fact, infection’s complications are mostly to ascribe to an hyperinflammatory systemic activation secondary to an overall dysregulated innate and adaptive immune response rather than to a direct cytopathic action of the virus [54, 63]. In particular, patients with severe antibodies deficiencies as XLA show a mild disease [64]. As opposed, some studies report an higher risk of severe infection in patients with primary immune deficit, particularly when the Interferons- type 1 (IFNs-1) pathway is involved [59, 60, 65–67]. Other case studies describe a greater risk for severe outcome among subjects with selected conditions, as Interleukin-1 receptor antagonist (DIRA) deficiency, and the mutations of STK4 and RAB27A [57]. Overall, the case-fatality ratio seems to vary deeply (being mainly influenced by the mean age and the type of PID) reaching peaks of nearly 10 times more than general population [61, 62]. These data may be representative of only the most severe PID and in addition the high rates of hospitalization are likely to simply reflect a greater facility in accessing hospital care. Moreover, the extreme phenotypic and clinical variability of PID (comprising over 450 different diseases with prognosis ranging from mild to severe/critical) doesn’t allow definitive conclusions extendable to the whole pediatric population. Literature provides contrasting evidence on the risk of severe SARS-CoV-2 infection in patients with PID. Age (older age calls for higher risk apart for the first year of life) and the presence of comorbidities seem to play an important role. In conclusion, for PID we retain appropriate to define the risk of progression to severe SARS-CoV-2 infection with a category based approach (Table 2) which may be useful in identifying patients to prioritize for mAbs administration [58].

**Table 2:** PIDs and risk of progression to severe COVID-19 (modified from: http://www.ukpin.org.uk/news-item/2020/03/24/covid-19-uk-pinupdate) [68]

Risk	Category	PID examples and/or molecular defect
High^a^	IFN-1 pathway defects PID with production of anti-IFN antibodies Combined PID Isolate/PID related CD4 lymphopenia (< 200/mmc) PID with deregulatory phenotype PID with anti-inflammatory phenotype uncontrolled by therapy^b^ Any PID [dependent on intra-venous immunoglobulin (IVIG) substitution therapy or antibiotic prophylaxis] associated to organ damage and/or chronic infection and/or malignancy Any PID [dependent on intra-venous immunoglobulin substitution therapy or antibiotic prophylaxis] on chronic oral corticosteroid or other immunosuppressant therapy Any PID which has undergone bone marrow transplant in the last 12 months Trisomy 21	TLR3, UNC93B1 STAT1, STAT2 APS1/APECED FHL, XIAP, RAB27A DIRA
Intermediate	XLA, CVI excluded from high risk PID Chronic Granulomatous Disease (CGD) Complement deficits	
Low	Minor antibodies deficiencies Minor complement deficiencies C1-inhibitor deficiency	IgA deficiency (selected cases) MBL deficiency, hereditary angioedema

^a^Examples of PID with at least one report of severe evolution in absence of significative comorbidities

^b^indirect evidence

Among acquired immunodeficiencies, **HIV** infection has a pivotal importance. Based on data from cohort studies and case series the risk for severe COVID-19 of these patients seems similar to that of the general population [69, 70]. However, an increased risk is described only for those with low lymphocyte count (CD4+ lymphocytes < 15% o < 200 cell/mm3) and severe comorbidities [71–73]. Thus, it should be reasonable to consent the use of mAbs in this subgroup of patients.

### Diabetes

**Diabetes** has been regarded as one of the major determinants of an unfavorable outcome in those infected by SARS-CoV-2 since the beginning of the pandemic [12]. To date, evidence in support of this thesis is mostly derived from adult-centered studies, focused on diabetes mellitus type 2 (DMT2) [74]. Studies including also the pediatric population confirm this trend, highlighting an enhanced risk for severe disease both in patients with DMT2 and in those with diabetes mellitus type 1 (DMT1) [75, 76]. Notably, an important multicenter study has remarked diabetic disease (in particular DMT1) as strongly related to a substantial increase in the risk for hospitalization (RR: 4.60; 95%CI: 3.91–5.62) and progression to severe disease in admitted patients (RR: 2.38; 95%CI: 2.06–2.76). It has to be taken into account the possibility of more severe presentation (and a consequent increase in the admission rate) driven by psychological and logistic factors. Still those evidence strongly supports the role of uncontrolled or complicated diabetes (both DMT1 and DMT2) as one of the major determinants of progression to severe SARS-CoV-2 disease in the pediatric age [14].

### Kidney disease

Although **chronic kidney failure** has already been defined as a determinant for severe SARS-CoV-2 infection in the adult, its role has still to be confirmed in the pediatric age [54]. In fact, the analysis carried out by the task force of the Italian Society for Pediatric Nephrology (SINePe) on the infections occurred between February and April 2020 in patients with nephropathies, does not show any increase both in the risk of infection and in the severity of disease. Still, to date, data for the pediatric age are largely based on fragmented reports of low reliability. In light of the solid studies on the adult patients and of the limited evidence in pediatric age, it may be appropriate to consent the use of anti-SARS-CoV-2 mAbs in those with chronic kidney failure, dependent upon chronic hemodialysis or peritoneal dialysis, together with those with immune deficit secondary to chronic immunosuppressant therapies [77, 78].

### Neurological and metabolic disorders

Epilepsy does not represent by itself a determinant for a greater risk of infection or for severe COVID-19 and vice-versa the infection does not call for an augmented risk of seizures. This seems to be equally true for neurofibromatosis type 1 (NF-1) patients, which are not to be regarded as high risk patients according to the existing evidences [79].

Subjects with neuromuscular conditions are considered at high risk for severe SARS-CoV-2 infection in presence of at least one of the following factors [80]:

- respiratory muscles dysfunction with forced vital capacity (FVC) < 60% (especially if associated to kyphoscoliosis);
- non-invasive ventilation or tracheostomy;
- oropharyngeal deficit with impaired cough and/or reduced respiratory clearance;
- hearth muscle involvement;
- metabolic conditions or impairment of the neuromuscular junctions at risk of deterioration in case of fever, fasting or infection;
- diabetes, obesity and or severe arterial hypertension;
- conditions at risk for rhabdomyolysis in case of fever, fasting or infection;
- chronic immunosuppressant or corticosteroid therapy.

In addition to the risk factors described above, an update of the British Paediatric Neurology Association (BPNA) of March 2021 indicates as at high risk for severe COVID-19 all the patients with neurological conditions with other comorbidities or immunosuppression (i.e., patients on immunosuppressants affected by Duchenne Muscular Dystrophy, West Syndrome, epileptic or neurodevelopmental encephalopathies, multiple sclerosis, tuberous sclerosis) and patients with respiratory impairment secondary to neurological conditions (i.e., infantile cerebral palsy, mitochondrial diseases, neuromuscular disease, leukodystrophy) [81].

Subjects affected by multiple sclerosis or similar demyelinating diseases targeting the central nervous system are apparently to be included in the “high risk” category based on a supposed greater incidence of pulmonary and extrapulmonary complications, driven by the neurological impairment and the altered immune reactivity. However, literature data show a risk for severe progression of SARS-CoV-2 infection similar to that of the general population [82].

Likewise, the use of mTOR inhibitors (e.g. Everolimus) in the treatment of tuberous sclerosis doesn’t seem related to a greater risk of infection neither to a more severe course of COVID-19 [83].

### Additional recommendations

Regarding mAbs administration in **genetic** and **polymalformative conditions** associated to significative comorbidities it is retained appropriate an individualized approach based on the guidance produced by the Genetic Alliance UK due to the substantial heterogeneity of this class of patients [84].

Anti-SARS-CoV-2 mAbs should be reserved to patients with at least one of the following conditions: severe intellectual disability, impaired respiratory clearance, bulbar impairment, neuromuscular comorbidities (e.g. severe spasticity/hypotonia, scoliosis or thoracic deformities) reduced level of consciousness.

Moreover, it should be useful to specify as various other diseases (i.e., severe malnutrition, anorexia) could represent potential risk factors for severe COVID-19, even if there are still no specific data. We thus retain appropriate an individualized approach under the guidance of the pediatrician in charge for the patient, reminding that the prescription of anti-SARS-CoV-2 mAbs in those patients is to be considered an off-label prescription.

Moreover, the Panel underlines as being immunized with an anti-SARS-CoV-2 vaccine does not contraindicate mAbs administration in the subjects at risk infected by COVID-19, regardless of the type of vaccine (m-RNA, adenoviral vector, etc.) received. Previous immunization must not influence future treatments, drugs’ and regimen’ choices included [85].

In conclusion, we report below (Table 3) the indications for mAbs administration in the pediatric patients. The Panel of experts from the Scientific Societies involved in this Consensus remarks that, to date, there are no sufficiently solid data regarding neither the higher risk of progression to severe COVID-19 infection nor the efficacy in preventing those progression with the use of anti SARS-CoV2 monoclonal antibodies in children with chronic diseases. Moreover, in the wide majority of cases, the most severe presentations in children and adolescents are represented by the so-called *Multisystem Inflammatory Syndrome in Children* (MIS-C) mostly occurring 3–6 weeks after SARS-CoV-2 infection in patients without significant comorbidities except, in some cases, obesity [86].

**Table 3:** Proposal of integration of the risk categories identified by AIFA for the administration of mAbs in subjects aged 12 to 17 years

Patients aged ≥12 years old, who tested positive for SARS-CoV-2, with mild-moderate disease, a recent onset (less than 10 days, except for patients with immunodeficiency and persistently positive molecular test but negative serologic test)* and the presence of risk factors for severe disease: a. BMI ≥ 95° percentile for age and gender (WHO Tables) b. Haemoglobinopathy (sickle cell anemia and thalassemia *major* or *intermedia*) c. Hereditary or acquired thrombophilia d. Onco-hematological patients with lymphopenia (< 300/mmc), neutropenia (< 500/mmc), high intensity treatment (AML, induction and reinduction phase for those with ALA, NHL, hematopoietic stem cell transplant recipients (< 30 days if autologous or < 100 days if allogenic) e. Solid organ or hematopoietic stem cells transplant recipients f. Congenital or acquired hearth diseases: single ventricle physiology or status post Fontan intervention (total cavo-pulmonary connection), severe heart valve disease, chronic cyanosis (SpO2 < 85%), severe ventricular dysfunction, therapy-dependent cardiomyopathies, pulmonary hypertension on treatment. g. Chronic obstructive or restrictive lung disease requiring daily therapy or biologics (e.g. severe or uncontrolled asthma dependent on specific monoclonal antibody therapy or oral steroids for symptoms control); cystic fibrosis with moderate to severe respiratory impairment, reduced BMI, transplant recipients or with other significative comorbidities; pulmonary fibrosis; bronchiolitis obliterans; bronchopulmonary dysplasia; chronic pulmonary GVHD h. Dependence on technological device (e.g. subjects with tracheostomy and/or gastrostomy) i. Inflammatory Bowel Diseases (IBDs) on immunosuppressant therapy j. PIDs at high risk for progression to severe disease (Table 2); case by case approach for those with mild-moderate risk PIDs k. Secondary Immunodeficiencies including HIV with low lymphocyte count (CD4+ lymphocytes < 15% or < 200/mmc) or with severe comorbidities, chemotherapy (< 6 months from suspension), hematopoietic stem cells transplant (< 3 months if autologous, < 6 months if allogenic or in presence of chronic active GVHD) or solid organ transplant and protracted immunosuppressant therapies l. Uncontrolled Diabetes mellitus (HbA1c > 9% or 75 mmol/mol) or with chronic complications m. Chronic renal failure requiring hemodialysis or peritoneal dialysis n. Neurological or neuro-muscular diseases with at least one of the following: - Respiratory muscles dysfunction with FVC < 60% (especially if associated with kyphoscoliosis) - Impaired cough and reduced respiratory clearance - Hearth involvement - Metabolic conditions or impairment of the neuromuscular junctions at risk of deterioration in case of fever, fasting or infection - Conditions at risk for rhabdomyolysis in case of fever, fasting or infection - Chronic immunosuppressant or corticosteroid therapies *Bamlanivimab+Etesevimab is approved for outpatients (not-hospitalized patients) without supplemental oxygen therapy for COVID 19; Casirivimab+Imdevimab is approved also for hospitalized patients and for patients with conventional oxygen therapy (excluding high flow and mechanical ventilation)	

Further, well powered multicenter studies are needed in order to better define, upon solid scientific basis, patients who may really benefit from those new mAbs administration, avoiding any inappropriate use.

## Data Availability

All the publication are listed in bibliography.
